# Characterization of Volatile Organic Compounds in Mango Ginger (*Curcuma amada* Roxb.) from Myanmar

**DOI:** 10.3390/metabo11010021

**Published:** 2020-12-30

**Authors:** Yanhang Chen, Musavvara Kh. Shukurova, Yonathan Asikin, Miyako Kusano, Kazuo N. Watanabe

**Affiliations:** 1 Graduate School of Life and Environmental Science, University of Tsukuba, Ibaraki 305-8572, Japan; yanhangchen@outlook.com (Y.C.); musavvara@gmail.com (M.K.S.); 2 Department of Bioscience and Biotechnology, Faculty of Agriculture, University of the Ryukyus, Okinawa 903-0213, Japan; y-asikin@agr.u-ryukyu.ac.jp; 3 Faculty of Life and Environmental Science, University of Tsukuba, Ibaraki 305-8572, Japan; kusano.miyako.fp@u.tsukuba.ac.jp; 4 Tsukuba-Plant Innovation Research Center, University of Tsukuba, Ibaraki 305-8572, Japan

**Keywords:** *Curcuma amada*, *Curcuma longa*, volatile organic compounds, chemical composition, GC-TOF-MS, Myanmar

## Abstract

*Curcuma amada* Roxb. (Zingiberaceae), commonly known as mango ginger because its rhizome and foliar parts have a similar aroma to mango. The rhizome has been widely used in food industries and alternative medicines to treat a variety of internal diseases such as cough, bronchitis, indigestion, colic, loss of appetite, hiccups, and constipation. The composition of the volatile constituents in a fresh rhizome of *C. amada* is not reported in detail. The present study aimed to screen and characterize the composition of volatile organic compound (VOC) in a fresh rhizome of three *C. amada* (ZO45, ZO89, and ZO114) and one *C. longa* (ZO138) accessions originated from Myanmar. The analysis was carried out by means of headspace solid-phase microextraction (HS-SPME) coupled with gas chromatography-time-of-flight-mass spectrometry (GC-TOF-MS). As a result, 122 VOCs were tentatively identified from the extracted 373 mass spectra. The following compounds were the ten most highly abundant and broadly present ones: *ar*-turmerone, α-zingiberene, α-santalene, (*E*)-γ-atlantone, cuparene, β-bisabolene, teresantalol, β-sesquiphellandrene, *trans*-α-bergamotene, γ-curcumene. The intensity of *ar*-turmerone, the sesquiterpene which is mainly characterized in *C. longa* essential oil (up to 15.5–27.5%), was significantly higher in *C. amada* accession ZO89 (15.707 ± 5.78^a^) compared to *C. longa* accession ZO138 (0.300 ± 0.08^b^). *Cis*-α-bergamotene was not detected in two *C. amada* accessions ZO45 and ZO89. The study revealed between-species variation regarding identified VOCs in the fresh rhizome of *C. amada* and *C. longa*.

## 1. Introduction

Plant volatile organic compounds (VOCs) are a relatively large group of plant secondary metabolites accumulated naturally and involved in plants’ interaction with their environment [1,2]. The emission of VOCs from plants has strong relevance to plant physiological processes, plant ecology, and atmosphere chemistry [3]. More than 1700 VOCs have been identified over the years from 90 different plant families [1]. They are divided into several classes including terpenoids, benzenoids and phenylpropanoids, fatty acid derivatives, amino acid derivatives, C5-branched compounds, and various nitrogen and sulfur-containing compounds in addition to a few species-/genus-specific compounds not represented in those major classes [4,5].

The genus *Curcuma* as one of the most important genera of the Zingiberaceae family comprises about 93–110 species with multiple uses as traditional medicine as well as in the modern industries [6,7]. The bioactive constituents in a rhizome of *Curcuma* species are related mostly to the non-volatile curcuminoids (curcumin, demethoxycurcumin, bisdemethoxycurcumin) and the volatile oil rich in sesquiterpenoids and monoterpenoids [6,8]. The phytochemical constituents of about 31 *Curcuma* species have been studied up to date [9], among which *C. longa* (turmeric) and *C. zedoaria* (zedoary) are the most well studied and reported [6,10].

*Curcuma amada* Roxb. is a perennial, rhizomatous herb growing up to about 80 cm in height and is morphologically similar to *Curcuma longa* species, known as turmeric. *C. amada* is distributed in the wild and cultivated in different parts of India, Myanmar, and Thailand [11]. In Myanmar, it is planted in the backyard and small-scale farming for domestic use and home consumption in the local markets. *C. amada* rhizome is the most valued part of the plant which contains a variety of terpenoids, flavonoids, and phenylpropanoids [6]. It is used not only as a food ingredient but also as a source of alternative medicine for treating digestive disorders such as indigestion, stomach pain, loss of appetite, constipation, colic, and hiccups, and to cure internally bronchitis, asthma, cough, and skin disorders, including itching and inflammation [8,11,12]. It has been used in the Ayurveda and Unani medicinal systems as a diuretic, laxative, appetizer, antipyretic, aphrodisiac, emollient, and expectorant [9].

Investigation of the phytochemical composition in *C. amada* has attracted increasing interest due to its nutritional and pharmacological significance [11]. More than 150 compounds have been reported in *C. amada* essential oil obtained by hydro- or steam distillation of the fresh or dry rhizome [9,13,14,15]. The VOCs in *C. amada* are vital, not merely due to their biological activity but are important both for taxonomic research and for understanding the interaction of plants with each other and with the environment [16]. However, studies are meager on the profiling of *C. amada* VOCs from the fresh rhizome. In addition, the taxonomic study of *C. amada* and *C. longa* remains difficult if only based on the morphological descriptions [11].

The metabolite profiling requires methods that facilitate the identification and quantification of hundreds of metabolites in a single or a complex plant mixture. Several approaches such as gas chromatography-mass spectrometry (GC-MS), liquid chromatography-mass spectrometry (LC-MS), gas chromatography time-of-flight mass spectrometry (GC-TOF-MS) have been established as highly accurate and sensitive methods for the analysis of highly complex mixtures of compounds [17]. Furthermore, the sample preparation techniques also play a vital role in achieving higher optimal results and obtaining consistent data in the later stages of analysis [18]. The simple and appropriate sample preparation method using small amounts of samples are expected for the precision and improvement of VOCs analysis. The headspace (HS) sampling method, as a solvent-free sample preparation technique, has been playing a fundamental role in studying the composition of the volatile fractions emitted from plants [19,20]. The solid-phase microextraction (SPME) technique has been proved for quick screening of VOCs in a wide range of products with the possibility to isolate trace compounds of different substrates [21,22]. Thus, the HS-SPME method can be used to extract multifarious volatile or semi-volatile organic compounds from a wide range of matrices, including air, water, and soil, which has been reported two decades ago [21]. Recently, the HS-SPME approach has widely been applied in the phytochemical study of plant species such as *Raphanus sativus*, *Brassica juncea* [23], *Cannabis sativa* L. among others [24]. 

The objective of the present study was the analysis of VOC profiles of three *C. amada* (ZO45, ZO89, and ZO114) and one *C. longa* (ZO138) accessions by means of HS-SPME coupled with GC-TOF-MS technique. The HS-SPME GC-TOF-MS approach can be a simple, fast, solvent-free, reproducible, and comprehensive metabolite profiling method for *Curcuma* species with a small amount of samples (50 µg/mL) to be used. *C. longa* has been used as a control sample for comparative characterization of the VOCs since phytochemical constituents of this species were better studied and reported [12]. To our current knowledge, this is the first report for the VOC profiling of *C. amada* using the HS-GC-TOF-MS analysis.

## 2. Results

### 2.1. VOC Profile of Curcuma spp. rhizomes and Principal Components Analysis (PCA) 

In total, 373 mass spectral (MS) peaks were obtained as a data matrix in the VOC profile of the 24 analyzed samples (three biological and two technical replicates for each accession). The obtained MS peaks were provisionally identified following Kusano et al. [19]. The similarity of each MS peaks and their retention indices (RIs) were compared with those reported in the corresponding libraries, i.e., the Flavors and Fragrance of Natural and Synthetic Compounds (FFNSC3) library, the Adams library [25], the Terpenoids library [26], the VocBinBase library [27], and the NIST14 [28]. As a result, 122 VOCs were tentatively identified comprising 32.7% of the rhizome volatile composition out of 373 detected mass spectra.

**Figure 1:**
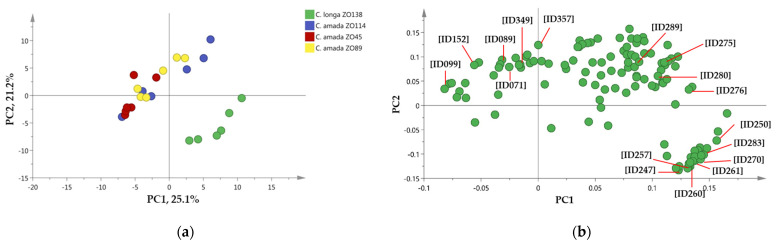
Principal component analysis (PCA) score (**a**) and loading (**b**) plots of the volatile organic compound (VOC) profiles of the *Curcuma amada* and *C. longa* samples. Principal components one and two (PC1, PC2) represent the first two principal components that account for a total of 46.3% of the variance. Loading plot indicated some representative compounds contributed to separation of the analyzed *Curcuma* species. Indicated compound IDs and names: [ID071], β-myrcene; [ID089], o-cymol; [ID099], 1,8-cineole; [ID152], camphor; [ID247], β-elemene; [ID250], α-funebrene; [ID257], α-santalene; [ID260], *trans*-α-bergamotene; [ID261], teresantalol; [ID270], α-santalol; [ID275], γ-curcumene; [ID276], cuparene; [ID280], α-zingiberene; [ID283], β-bisabolene; [ID289], β-sesquiphellandrene; [ID349], ar-turmerone; [ID357], (*Z*)-γ-atlantone.

The multivariate data analysis, i.e., principal component analysis (PCA), of the obtained data was performed on SIMCA 14.0 in order to reduce the dimensionality of the multivariate data and visualize similarities and differences in the VOC composition of four *Curcuma* species [29]. The 122 identified VOCs were included in the PCA analysis (Figure 1) with the first two components retaining 25.1% and 21.2% of the total variance in the data set. 

The PCA score plot of the VOC profile data showed species-dependent differentiation resulting from the separation group of *C. amada* samples from a group of *C. longa* samples in the different quadrants of the generated plot (Figure 1a). The group of *C. amada* samples—accessions ZO45, ZO89, and ZO114—was scattered between lower and upper negative/positive quadrants, while the group of *C. longa* samples—accession ZO138—was situated in a lower negative quadrant of the plot (Figure 1a).

The distribution of the identified major VOCs in the profiles of *C. amada* and *C. longa* accessions was investigated by performing the PCA loading plot. Among the annotated VOCs some were highlighted in the PCA loading plot as representative compounds that contributed to the separation of the two species, i.e., *C. amada* and *C. longa* (Figure 1b). In *C. amada* samples, the compounds such as β-myrcene [ID071], o-cymol [ID089], 1,8-cineole [ID099], camphor [ID152], γ-curcumene [ID275], cuparene [ID276], α-zingiberene [ID280], β-sesquiphellandrene [ID289], *ar*-turmerone [ID349], and (*E*)-γ-atlantone [ID357] were detected at relatively high level (Figure 1b, Table S1). The VOCs like β-elemene [ID247], α-funebrene [ID250], α-santalene [ID257], *trans*-α-bergamotene [ID260], teresantalol [ID261], α-santalol [ID270], and β-bisabolene [ID283] were found in abundance in *C. longa* sample (Figure 1b). The result was consistent with significant differences between these species as shown in Supplementary Table S1.

### 2.2. Major Discriminative VOCs Detected in C. amada and C. longa Species

We compared the metabolite profile of three *C. amada* and one *C. longa* accessions to investigate whether the VOC composition of these two closely related species can be used in their differentiation. The significant differences of tentatively identified VOCs in the HS of samples were analyzed by Tukey’s HSD test from the average normalized value of six replicates per sample. The similarities and differences were observed regarding identified VOCs within *C. amada* accessions, as well as between *C. amada* and *C. longa* accessions (Supplementary Table S1). In total, 74 VOCs were significantly different, out of which 30 compounds were found to be significantly different between *C. amada* and *C. longa* accessions, and 54 compounds were significantly different among three *C. amada* accessions, respectively (Supplementary Table S1). One compound *cis*-α-bergamotene was not detected in *C. amada* accessions ZO45 and ZO89. The degree of the VOC changes was correlated with the result obtained by PCA analysis (Figure 1b).

## 3. Discussion

The identification of the VOC profile in the rhizomes of three *C. amada* (ZO45, ZO89 and ZO114) and *C. longa* (ZO138) accessions were carried out by application of the non-targeted GC-TOF-MS method. Each compound was identified by comparing its RIs, determined with respect to a homologous series of normal alkanes, and by matching the recorded mass spectra of each compound by the degree of its similarity with those reported in the commercial libraries, allowing to detect qualitative differences between the analyzed samples. In total, 122 VOCs were identified in the VOC profile of two studied *Curcuma* species. The result corresponds with those reported by Qudah et al. [30] that more than 130 chemical constituents have been reported in the rhizome of *C. amada*, but only 121 compounds have been tentatively identified.

The experimental plant materials used in the present study were cultivated under similar conditions in order to exclude possible variability in the metabolite profile due to cultivation factors. However, the VOC profiles of the three *C. amada* and one *C. longa* genotypes varied. The result of Tukey’s test showed that 54 compounds were significantly different among the three examined *C. amada* accessions (ZO45, ZO89, and ZO114) and 30 compounds were significantly different in *C. longa* accession ZO138 (Supplementary Table S1). The major VOCs detected to be significantly different between *C. amada* and *C. longa* accessions were α-santalene, β-caryophyllene, trans-α-bergamotene, teresantalol, cuparene, β-bisabolene, (*E*)-γ-bisabolene, (*Z*)-α-bisabolene, and α-funebrene. The VOC profile of accession ZO45 was leading by compounds 1,8-cineole, α-humulene, α-zingiberene, β-sesquiphellandrene, and β-caryophyllene as major ones. The main constituents in the VOC profile of accessions ZO89 and ZO114 were α-zingiberene, (*E*)-γ-atlantone (only in ZO89), β-sesquiphellandrene, cuparene, following by 2,4-di-tert-butylphenol, γ-curcumene, and β-caryophyllene. The sesquiterpene *cis*-α-bergamotene was not detected in the volatile profile of accessions ZO45 and ZO89. Our results were inconsistent with those reported in several studies [8,9,31], whereas myrcene (80.5–88.8%) following by camphor (5.5–17.9%) and *ar*-curcumene (up to 28.1%) were reported as major constituents (in essential oil obtained from the fresh or dry rhizome) characterized for *C. amada*. 

In the present study, α-zingiberene, β-sesquiphellandrene, and cuparene were the major compounds characterized in the VOC profile of three *C. amada* accessions, and α-santalene, cuparene, β-bisabolene, and teresantalol were the main compounds in the VOC profile of *C. longa* accession from Myanmar. The certain volatile compounds were inherent and special only in distinctive *Curcuma* species. For instance, *ar*-turmerone (57.48%) is the main compound characteristic of *C. longa* rhizome oil [9,32]; curdione (50.6%), camphor (35.8%), camphene (10.2%), and 1,8-cineole (10.1%) are reported as major constituents in rhizome oil of *C. aromatica* [9,10]; *C. zedoaria* rhizome oil is consisted mainly of 1,8-cineole (38.4%), epicurzerene (29.4%), and α-copaene (17.4%) as a major one among the others [6]. Our result showed that the intensity of *ar*-turmerone was high in *C. amada* accession ZO89 and was significantly different compared to *C. longa* or two other *C. amada* accessions. It is well-known that the extent of each volatile constituent varies depending on species/cultivars and cultivation conditions such as weather, irrigation, soil type, pests. We suggest the observed variation probably was related to the defensive function of VOCs, which improves plant resistance efficiency either to biotic or abiotic stresses.

*C. amada* and *C. longa* are closely related species. It is difficult to distinguish solely by their morphological characters. Therefore, for contribution, evaluation, standardization, and promotion of plant species, the assessment of chemical constituents is vital for the taxonomic study and the quality control of herbal medicines [33].

### 3.1. The VOCs Possessing Therapeutic Effect

It is apparent that many of the identified substances are reported to possess various potentially biological activities in *Curcuma* species. The major compounds detected in this study that were previously reported [13,14,34] with therapeutic effect were α-zingiberene and *ar*-turmerone. The *ar*-turmerone as a natural antibiotic possess antioxidant, anti-inflammatory, anti-tumor, and immune-activating properties [35]; it is used as an insecticidal and repellent agent in crop industries [36], as a potent anti-venom against snakebites in traditional medicine, and as an agent to enhance stem-cell proliferation [35]. The *cis*-α-bergamotene was detected in *C. longa* and *Zingiber officinale* (common ginger), contributing to the antioxidant and antimicrobial properties [37,38]. It was also reported in the plant defense studies as one of the most abundant VOC in the HS of high-nitrogen and low-nitrogen plants and the release of which was not affected by nitrogen limitation [39,40]. The monoterpene *o*-cymol reported in the essential oils of several species has antifungal activity [41,42,43,44]. The compound α-humulene (α-caryophyllene), reported in other *Curcuma* species, has the antimicrobial activity [45,46]. The γ-curcumene was mainly reported in *C. longa* as an antioxidant agent [37,47]. Furthermore, 2,4-di-tert-butylphenol was reported as a promising compound with antioxidant and antifungal activities [48]. Beta-sesquiphellandrene has antioxidant and antimicrobial activities reported in other Zingiberaceae species [49,50]. 

### 3.2. The VOCs Possibly Contributing to the Mango Aroma

*C. amada* commonly known as the “mango ginger” or “manga manjal” [9] due to its aroma of raw mango and many attempts were made to identify the compounds contributing to this notable feature. The compounds pinene, δ-3-carene, (*Z*)-β-ocimene, and myrcene (β-myrcene) were reported [31,51] to contribute for the characteristic mango odor in *C. amada* rhizome. We did not detect these compounds in the current study, although the assessed *C. amada* accessions displayed light mango aroma. Whilst compounds such as hexanal, beta-myrcene, terpinolene, nonanal, (*E*)-2-nonenal, decanal, and beta-caryophyllene reported as key contributors to the mango aroma in mango fruits [52,53,54], we presume the above-mentioned compounds may also contribute to the mango aroma in studied *C. amada* accessions.

## 4. Materials and Methods 

### 4.1. Plant Materials

The rhizomes of the three *C. amada* (ZO45, ZO89, and ZO114) and one *C. longa* (ZO138) accessions were used as plant materials to conduct the current study. *Curcuma* species were obtained from Myanmar under the Myanmar–Japan cooperative project for the exploration and utilization of the plant genetic resources in Myanmar. The plant material was transferred to Japan via the Standard Material Transfer Agreement (SMTA) under the International Treaty on Plant Genetic Resources for Food and Agriculture (ITPGRFA), the United Nations Food and Agriculture Organization (UN FAO) [55]. The candidate rhizomes were planted in plastic pots of 30 × 50 cm (diameter × height) containing 2–3 cm of big granular soil (Akadama Churyu, Kato Sangyo Co., Nishinomiya, Japan) at the bottom of pot and ready to use a pre-mixed soil (Hana to Yasai no Bayo Do, Kato Sangyo Co., Nishinomiya, Japan) as a growth medium. Pots were arranged in the experimental field of the Gene Research Center, University of Tsukuba, Japan (36°6′0 N latitude and 140°6′0 E longitude). The plant materials were grown in three biological replications and were fertilized every four weeks by liquid fertilizer (HYPONeX Co., Ltd., Osaka, Japan) consisting of nitrogen, phosphorus, and potassium in the ratio of 6:10:5 (N:P:K). Table 1 represents information regarding plant material accession number, origin and source of plant material, and year of acquisition.

### 4.2. Sample Preparation and Experimental Design

The *Curcuma* rhizomes were harvested on 17 October 2018, when the night/day temperature was +15–+20 °C. The collected rhizomes were carefully washed and cleaned under tap water to remove soil residues, and dried in a paper towel in dark condition for approximately 30 min. Then, rhizomes were chopped into slices of 1.0 cm using stainless steel surgical blades (Feather Safety Razor Co., Ltd., Osaka, Japan). About 15 g of chopped rhizome samples were transferred to a homogenization tube and stored at −80 °C for future processing. The rhizomes pieces were cryohomogenized into powder in a Multi-beads Shocker MB2000 (Yasui Kikai Co., Ltd., Osaka, Japan) at 2800 rpm for 16 s.

The experiment was designed and conducted in three biological and two technical replications per accession.

### 4.3. Analytical Conditions and HS Collection

All chemicals and reagents used in this study were of analytical grade. The EPA 524.2 fortification solution (20 μg/mL of fluorobenzene, 4-bromofluorobenzene, and 1,2-dichlorobenzene-*d_4_*) (Sigma-Aldrich, Tokyo Japan) was used as an internal standard (IS). The n-alkane standard solution purchased from Fluka Chemical (Tokyo, Japan) was used for the determination of the RI (C8–C20). The EDTA solution in a concentration of 100 mM (pH 7.5) was used to inhibit enzymatic activity [1].

The 10 mg of frozen powder of each sample was weighed and dissolved in 1 mL of distilled water to prepare a stock solution. The stock solution of 10 mg/mL concentration was diluted up to 50 µg/mL of working solution and stored in −30 °C up to date of analysis. The working aliquots were sonicated for 10 min using the Branson sonicator (Branson Ultrasonics, CT, USA) before analytes for VOC extraction. The samples were prepared in 20 mL headspace Supelco vials (Missouri, USA) consisted of 1 mL of EDTA solution, 1 mL of the sample solution, and 10 μL of IS. 

The preconditioned 50/30 μm solid-phase microextraction (SPME) fiber assembly DVB/CAR/PDMS purchased from Supelco (Cassopolis, MI, USA) was used to extract VOCs from samples HS. The extracted analytes were sampled through CTC CombiPAL auto-sampler (CTC Analytics, Zwingen, Switzerland), purchased from AMR (Tokyo, Japan).

### 4.4. GC-TOF-MS Analysis

The collected volatile fractions were injected in splitless mode into a 6890N Agilent GC (Agilent Technologies, Wilmington, NC, USA), which was equipped with a Rxi-5Sil MS fused-silica capillary column (30 m × 0.25 mm inner diameter × 0.25 μm, RESTEK, Bellefonte, PA, USA). Helium was used as carrier gas at a constant flow rate of 1.0 mL/min. The programmed GC oven temperature started at 55 °C for 3 min, then increased to 150 °C at the rate of 15 °C/min, then increased to 200 °C at the rate of 3 °C/min with a final hold at 200 °C for 2 min. The back inlet temperature was kept at 250 °C. The mass spectral analysis was done on time-of-flight mass spectrometer Pegasus III 4D TOF-MS (LECO, MI, USA). The mass spectral ionization energy was 70 eV with an ionization source temperature set on 200 °C. The MS scan range (*m*/*z*) was 29–500 amu with an acquisition rate of 30 spectra/s.

### 4.5. Data Processing

The data processing and provisional VOC identification were done according to Kusano et al. [19] with modifications. The non-processed MS data were transformed into the NetCDF format using Leco ChromaTOF version 4.71.0.0 (LECO, St. Joseph, MI, USA) and MATLAB 7.0 (Mathworks, Natick, MA, USA). The data-pretreatment procedures such as baseline correction, smoothing, peak alignment, time-window setting, and deconvolution by the hierarchical multi-curve resolution (H-MCR) were performed on MATLAB 7.0 (Mathworks, Natick, MA, USA) [56]. 

The adjusted mass spectra obtained by the H-MCR method [57] were matched against those reference mass spectra reported in the commercial libraries by using the NIST version 2.0 mass spectral search program and the customized peak annotation software following Kusano et al. [19]. The referenced libraries used for the VOC identification were Wiley’s FFNSC3 library, the Adams library [25], the Terpenoids library [26], the VocBinBase Library [27], and the NIST14 [28].

The compounds were identified according to the guideline for metabolite identification [56]. To identify similar or very similar compounds from the referenced libraries, the similarity index greater than ≥800 with the RI differences less than <30 unit was applied. When the standard deviation (SD) of the absolute RI difference between the compounds was less than 8.8 units, a similarity of ≥799 with differences less than 20 units were applied to determine whether peaks were from a putatively annotated compound.

### 4.6. Statistical Analysis

The SIMCA 14.0 software (Umetrics AB, Umeå, Sweden) and SPSS 22.0 (SPSS Inc., Chicago, IL, USA) were used for multivariate data analysis. The PCA was conducted on SIMCA software. The analysis of variance (ANOVA) was run using the SPSS software with means of six replicates per sample and significant differences (*p* ≥ 0.05) were calculated by Tukey’s HSD test. 

## 5. Conclusions

The fresh rhizomes of three mango ginger (*C. amada*) accessions (ZO45, ZO89, ZO114) in comparison with one turmeric (*C. longa*) accession (ZO138) from the collection of the GRC UT were analyzed by GC-TOF-MS. A total of 122 VOCs were tentatively identified, of which 73 compounds were significantly different and cause of observed within/between-species variation. α-Santalene, β-caryophyllene, trans-α-bergamotene, teresantalol, cuparene, β-bisabolene, (E)-γ-bisabolene, (Z)-α-bisabolene, and α-funebrene were major VOCs contributing to the differentiation of *C. amada* and *C. longa* genotypes. Major VOCs contributed variation within *C. amada* genotypes were 1,8-cineole, α-humulene, α-zingiberene, β-sesquiphellandrene, β-caryophyllene, β-sesquiphellandrene, γ-curcumene, β-curcumene, γ-amorphene, β-caryophyllene, ar-turmerone, and (E)-γ-atlantone. The significant differences regarding identified VOCs among three *C. amada* accessions were higher compared with differences detected between *C. amada* and *C. longa* accessions. In total, 54 compounds among three *C. amada* samples and 30 compounds between *C. amada* and *C. longa* samples were significantly different. The result reflects the existing chemical variation within each genotype rather that each genotype is a separate chemotype. Considering the utilization of the plant and the plant-based alternative products used in ethnic medicine of Myanmar, *C. amada* can also be a potential resource for future research with ample medicinal, pharmaceutical, and chemical applicability. The geographical distribution of *C. amada* species can influence not only the morphological features but also the presence or absence of secondary metabolites, their amount in different tissues (rhizome, steam, leaf, flower), genotypes, and populations. This is a considerable gap for future research in this direction.

## Figures and Tables

**Table 1:** List of *C. amada* and *C. longa* species with indicated accessions’ number, country of origin, source, and year of acquisition with the Standard Material Transfer Agreement (SMTA).

Accession No.	Species	Country of Origin	Source	Year of Acquisition with SMTA
ZO45	*C. amada*	Myanmar	Genebank	2004
ZO89	*C. amada*	Thailand ^1^	Rural market	2005
ZO114	*C. amada*	Myanmar	Local farm	2004
ZO138	*C. longa*	Myanmar	Local farm	2006

^1^ Thailand: The material is confirmed that it probably was exported from Myanmar, which means its origin is Myanmar.

## Data Availability

The data presented in this study are available on request from the corresponding author. The data are not publicly available due to the Institutional regulations at UT.

## Supplementary Materials

The following are available online at https://www.mdpi.com/2218-1989/11/1/21/s1, Table S1: The composition of VOCs in the rhizome of *C. amada* (ZO45, ZO89, ZO114) and *C. longa* (ZO138) accessions, and the significant differences calculated between them based on Tukey’s HSD test.
