# Supplementary material for: Characterization of Volatile Organic Compounds in Mango Ginger (Curcuma amada Roxb.) from Myanmar
Source: Metabolites. 2020 Dec 30;11(1):21. doi: 10.3390/metabo11010021 (PMC7824228; doi:10.3390/metabo11010021)
Supplement: Supplementary file 1 [file metabolites-11-00021-s001.pdf]

**Supplementary Material:** Characterization of Volatile Organic Compounds in Mango Ginger (*Curcuma amada* Roxb.) from Myanmar by Yanhang Chen, Musavvara Kh. Shukurova, Yonathan Asikin, Miyako Kusano and Kazuo N. Watanabe

**Table S1.** The composition of VOCs in the rhizome of *C. amada* (ZO45, ZO89, ZO114) and *C. longa* (ZO138) accessions, and the significant differences calculated between them based on Tukey's HSD test.

| Volatile Organic Compounds | ZO45 | ZO89 | ZO114 | ZO138 |
| --- | --- | --- | --- | --- |
| 1,3,5-Cycloheptatriene | 0.015±0.01c | 0.047±0.02a | 0.039±0.02b | 0.026±0.01ab |
| 1,2-Diethylcyclobutane | 0.004±0.00$^{a}$ | 0.001±0.00$^{b}$ | 0.002±0.00$^{ab}$ | 0.002±0.00$^{b}$ |
| 2-Methyl-2-heptene | 0.003±0.00$^{bc}$ | 0.001±0.00$^{c}$ | 0.008±0.01$^{a}$ | 0.006±0.00$^{ab}$ |
| Ethylbenzene | 0.005±0.00$^{b}$ | 0.008±0.00$^{a}$ | 0.008±0.00$^{ab}$ | 0.008±0.00$^{ab}$ |
| Butyrolactone | 0.006±0.00$^{a}$ | 0.000±0.00$^{b}$ | 0.002±0.00$^{ab}$ | 0.004±0.00$^{ab}$ |
| β-Myrcene | 0.001±0.00$^{ab}$ | 0.002±0.00$^{a}$ | 0.000±0.00$^{b}$ | 0.000±0.00$^{b}$ |
| 2,2,4,6,6-Pentamethylheptane | 0.009±0.00$^{b}$ | 0.009±0.00$^{b}$ | 0.016±0.01$^{a}$ | 0.010±0.00$^{ab}$ |
| Octanal | 0.006±0.00$^{b}$ | 0.009±0.00$^{ab}$ | 0.011±0.00$^{a}$ | 0.007±0.00$^{b}$ |
| α-Phellandrene | 0.001±0.00$^{b}$ | 0.066±0.04$^{a}$ | 0.000±0.00$^{b}$ | 0.000±0.00$^{b}$ |
| *o*-Cymol | 0.014±0.03$^{b}$ | 0.064±0.03$^{a}$ | 0.001±0.00$^{b}$ | 0.001±0.00$^{b}$ |
| 4-Methyl-2-propylpentanol | 0.108±0.03$^{b}$ | 0.168±0.05$^{a}$ | 0.169±0.07$^{a}$ | 0.127±0.03$^{ab}$ |
| 1,8-Cineole | 1.671±0.90$^{a}$ | 0.252±0.13$^{b}$ | 0.334±0.11$^{b}$ | 0.079±0.03$^{b}$ |
| 3,3,6-Trimethyl-1,5-heptadien-4-one | 0.001±0.00$^{b}$ | 0.003±0.00$^{a}$ | 0.004±0.00$^{a}$ | 0.003±0.00$^{a}$ |
| 1-Octanol | 0.004±0.00$^{b}$ | 0.003±0.00$^{b}$ | 0.005±0.00$^{a}$ | 0.004±0.00$^{b}$ |
| 2-Nonanone | 0.005±0.00$^{b}$ | 0.001±0.00$^{b}$ | 0.047±0.01$^{a}$ | 0.000±0.00$^{b}$ |
| 3-Methylbutyl-2-hydroxypropionate | 0.042±0.02$^{a}$ | 0.002±0.00$^{b}$ | 0.054±0.01$^{a}$ | 0.001±0.00$^{b}$ |
| Camphor | 0.023±0.01$^{a}$ | 0.004±0.00$^{b}$ | 0.019±0.00$^{a}$ | 0.003±0.00$^{b}$ |
| (*E*)-2-Nonenal | 0.001±0.00$^{ab}$ | 0.004±0.00$^{a}$ | 0.003±0.00$^{ab}$ | 0.000±0.00$^{b}$ |
| Isoborneol | 0.019±0.01$^{a}$ | 0.000±0.00$^{b}$ | 0.005±0.00$^{b}$ | 0.000±0.00$^{b}$ |
| Borneol | 0.008±0.00$^{a}$ | 0.002±0.00$^{b}$ | 0.003±0.00$^{b}$ | 0.002±0.00$^{b}$ |
| Menthol | 0.002±0.00$^{c}$ | 0.004±0.00$^{b}$ | 0.005±0.00$^{a}$ | 0.004±0.00$^{b}$ |
| 4-Terpineol | 0.007±0.00$^{a}$ | 0.002±0.00$^{b}$ | 0.002±0.00$^{b}$ | 0.001±0.00$^{b}$ |
| α-Terpineol | 0.049±0.02$^{a}$ | 0.023±0.01$^{b}$ | 0.032±0.01$^{b}$ | 0.022±0.00$^{b}$ |
| Decanal | 0.011±0.00$^{b}$ | 0.015±0.00$^{ab}$ | 0.018±0.01$^{a}$ | 0.012±0.00$^{b}$ |
| 2,7,10-Trimethyldodecane | 0.001±0.00$^{b}$ | 0.001±0.00$^{b}$ | 0.002±0.00$^{b}$ | 0.008±0.00$^{a}$ |
| Sesquithujene | 0.003±0.00$^{c}$ | 0.006±0.00$^{b}$ | 0.006±0.00$^{b}$ | 0.013±0.00$^{a}$ |
| β-Elemene | 0.000±0.00$^{b}$ | 0.000±0.00$^{b}$ | 0.000±0.00$^{b}$ | 0.023±0.00$^{a}$ |
| α-Funebrene | 0.016±0.01$^{c}$ | 0.043±0.02$^{b}$ | 0.047±0.02$^{b}$ | 0.116±0.01$^{a}$ |
| Cyperene | 0.000±0.00$^{b}$ | 0.000±0.00$^{b}$ | 0.000±0.00$^{b}$ | 0.006±0.00$^{a}$ |
| *cis*-α-Bergamotene | ND$^{b}$ | ND$^{b}$ | 0.000±0.00$^{b}$ | 0.053±0.01$^{a}$ |
| α-Santalene | 0.000±0.00$^{b}$ | 0.000±0.00$^{b}$ | 0.000±0.00$^{b}$ | 5.573±0.80$^{a}$ |

| Volatile Organic Compounds | ZO45 | ZO89 | ZO114 | ZO138 |
|---|---|---|---|---|
| β-Caryophyllene | 0.353±0.31$^{b}$ | 0.515±0.24$^{ab}$ | 0.075±0.03$^{c}$ | 0.646±0.15$^{a}$ |
| *trans*-α-Bergamotene | 0.016±0.01$^{b}$ | 0.024±0.01$^{b}$ | 0.019±0.01$^{b}$ | 1.239±0.17$^{a}$ |
| Teresantalol | 0.000±0.00$^{b}$ | 0.000±0.00$^{b}$ | 0.000±0.00$^{b}$ | 2.243±0.43$^{a}$ |
| β-Farnesene | 0.032±0.02$^{c}$ | 0.121±0.04$^{b}$ | 0.054±0.02$^{c}$ | 0.411±0.08$^{a}$ |
| β-Santalene | 0.000±0.00$^{b}$ | 0.000±0.00$^{b}$ | 0.000±0.00$^{b}$ | 0.426±0.09$^{a}$ |
| α-Santalol | 0.005±0.00$^{b}$ | 0.025±0.01$^{b}$ | 0.036±0.02$^{b}$ | 0.341±0.07$^{a}$ |
| α-Humulene | 0.597±0.57$^{a}$ | 0.059±0.03$^{b}$ | 0.041±0.02$^{b}$ | 0.070±0.01$^{b}$ |
| α-Curcumene | 0.021±0.02$^{b}$ | 0.033±0.02$^{ab}$ | 0.051±0.02$^{a}$ | 0.055±0.01$^{a}$ |
| γ-Curcumene | 0.252±0.20$^{c}$ | 0.517±0.28$^{b}$ | 0.886±0.39$^{a}$ | 0.625±0.11$^{ab}$ |
| Cuparene | 1.269±1.10$^{b}$ | 2.046±0.94$^{ab}$ | 2.328±1.00$^{ab}$ | 2.912±0.73$^{a}$ |
| Germacrene D | 0.003±0.00$^{b}$ | 0.009±0.01$^{b}$ | 0.011±0.01$^{b}$ | 0.557±0.07$^{a}$ |
| α-Zingiberene | 1.945±1.54$^{c}$ | 3.051±1.82$^{bc}$ | 5.815±2.63$^{a}$ | 4.389±0.63$^{b}$ |
| β-Bisabolene | 0.292±0.23$^{b}$ | 0.401±0.22$^{b}$ | 0.648±0.31$^{b}$ | 2.324±0.48$^{a}$ |
| β-Curcumene | 0.075±0.06$^{c}$ | 0.265±0.13$^{b}$ | 0.353±0.14$^{b}$ | 0.557±0.10$^{a}$ |
| (*Z*)-γ-Bisabolene | 0.001±0.00$^{b}$ | 0.008±0.01$^{b}$ | 0.004±0.00$^{b}$ | 0.086±0.04$^{a}$ |
| β-Sesquiphellandrene | 1.107±0.84$^{b}$ | 2.359±1.23$^{a}$ | 2.815±1.12$^{a}$ | 2.126±0.35$^{ab}$ |
| γ-Amorphene | 0.049±0.09$^{b}$ | 0.179±0.15$^{ab}$ | 0.209±0.19$^{ab}$ | 0.296±0.09$^{a}$ |
| (*E*)-γ-Bisabolene | 0.026±0.02$^{c}$ | 0.098±0.06$^{b}$ | 0.075±0.04$^{bc}$ | 0.303±0.05$^{a}$ |
| (*Z*)-α-Bisabolene | 0.001±0.00$^{c}$ | 0.023±0.01$^{b}$ | 0.009±0.00$^{bc}$ | 0.097±0.03$^{a}$ |
| 4-Hydroxy-3a,7a-dimethyl-4,5-dihydro-3H-2-benzofuran-1-one | 0.003±0.00$^{b}$ | 0.016±0.00$^{a}$ | 0.004±0.00$^{b}$ | 0.006±0.00$^{b}$ |
| 3,7(11)-Selinadiene | 0.000±0.00$^{b}$ | 0.000±0.00$^{b}$ | 0.000±0.00$^{b}$ | 0.012±0.00$^{a}$ |
| Dodecanoic acid | 0.003±0.00$^{b}$ | 0.019±0.01$^{a}$ | 0.013±0.01$^{ab}$ | 0.009±0.01$^{ab}$ |
| (*Z*)-Nerolidol | 0.000±0.00$^{b}$ | 0.010±0.01$^{a}$ | 0.000±0.00$^{b}$ | 0.000±0.00$^{b}$ |
| 1,2,3,1',2',3'-Hexamethyl-bicyclopentyl-2,2'-diene | 0.002±0.00$^{b}$ | 0.028±0.01$^{a}$ | 0.006±0.00$^{b}$ | 0.002±0.00$^{b}$ |
| Santalenone | 0.003±0.00$^{b}$ | 0.006±0.01$^{b}$ | 0.006±0.00$^{b}$ | 0.297±0.10$^{a}$ |
| 1-[2-(Isobutyryloxy)-1-methylethyl]-2,2-dimethylpropyl 2-methylpropanoate | 0.097±0.11$^{b}$ | 0.057±0.01$^{b}$ | 0.109±0.07$^{b}$ | 0.271±0.05$^{a}$ |
| Thujopsan-2-α-ol | 0.000±0.00$^{b}$ | 0.000±0.00$^{b}$ | 0.000±0.00$^{b}$ | 0.070±0.02$^{a}$ |
| Curlone | 0.001±0.00$^{b}$ | 0.039±0.01$^{b}$ | 0.010±0.00$^{b}$ | 0.301±0.07$^{a}$ |
| Helifolen-12-al A | 0.016±0.01$^{c}$ | 0.283±0.07$^{a}$ | 0.049±0.01$^{c}$ | 0.129±0.01$^{b}$ |
| β-Eudesmol | 0.013±0.01$^{a}$ | 0.000±0.00$^{b}$ | 0.000±0.00$^{b}$ | 0.000±0.00$^{b}$ |
| Bicyclo[3.2.2]non-8-ene-6,7-dicarboxylicanhydride | 0.001±0.00$^{b}$ | 0.035±0.01$^{a}$ | 0.006±0.00$^{b}$ | 0.002±0.00$^{b}$ |
| γ-Eudesmol | 0.000±0.00$^{b}$ | 0.012±0.00$^{a}$ | 0.000±0.00$^{b}$ | 0.001±0.00$^{b}$ |
| α-Acorenol | 0.001±0.00$^{ab}$ | 0.000±0.00$^{b}$ | 0.001±0.00$^{a}$ | 0.001±0.00$^{a}$ |
| (*E*)-Sesquilavandulol | 0.000±0.00$^{b}$ | 0.000±0.00$^{b}$ | 0.000±0.00$^{b}$ | 0.011±0.00$^{a}$ |
| *ar*-Turmerone | 0.111±0.24$^{b}$ | 15.707±5.78$^{a}$ | 0.622±1.30$^{b}$ | 0.300±0.08$^{b}$ |
| (*Z*)-γ-Atlantone | 0.005±0.01$^{b}$ | 0.032±0.02$^{a}$ | 0.011±0.01$^{b}$ | 0.004±0.00$^{b}$ |
| Germacrone | 0.000±0.00$^{b}$ | 0.000±0.00$^{b}$ | 0.000±0.00$^{b}$ | 0.187±0.06$^{a}$ |

| Volatile Organic Compounds | ZO45 | ZO89 | ZO114 | ZO138 |
|---|---|---|---|---|
| (*E*)-γ-Atlantone | 0.039±0.07$^{b}$ | 3.169±1.43$^{a}$ | 0.069±0.16$^{b}$ | 0.016±0.04$^{b}$ |
| (6*S*,7*R*)-Bisabolone | 0.004±0.00$^{c}$ | 0.039±0.01$^{a}$ | 0.018±0.01$^{b}$ | 0.011±0.00$^{bc}$ |
| (*E*)-α-Atlantone | 0.008±0.00$^{b}$ | 0.051±0.01$^{a}$ | 0.014±0.01$^{b}$ | 0.011±0.01$^{b}$ |
| Octadecane | 0.000±0.00$^{b}$ | 0.003±0.00$^{a}$ | 0.001±0.00$^{b}$ | 0.000±0.00$^{b}$ |
| Methyl-15-methylhexadecanoate | 0.001±0.01$^{b}$ | 0.004±0.00$^{b}$ | 0.009±0.00$^{a}$ | 0.003±0.00$^{b}$ |

Compounds with the same letter are not significantly different (Tukey’s HSD test, $p < 0.05$). Each value shows the average ± standard deviation (std) of six replications (two biological and three technical replications) for each examined *Curcuma* species. The values which was not found (equal to 0.000) were excluded from the table.
